# A randomized controlled trial of daily weighing in pregnancy to control gestational weight gain

**DOI:** 10.1186/s12884-020-02884-1

**Published:** 2020-04-16

**Authors:** Christopher Arthur, Ellen Di Corleto, Emma Ballard, Alka Kothari

**Affiliations:** 1 Griffith University, Southport, Queensland Australia; 2 University of Queensland, St Lucia, Queensland Australia; 3 Redcliffe Hospital, Anzac Avenue, Redcliffe, Queensland Australia; 4 QIMR Berghofer Medical Research Institute, Brisbane, Queensland Australia

**Keywords:** Pregnancy, Weight gain, Randomized controlled trial, Australia, Human

## Abstract

**Background:**

Excessive gestational weight gain is a modifiable risk factor for the development of obstetric and neonatal complications, and can have a lifelong impact on the health of both mother and offspring. The purpose of this study was to assess whether in addition to standardized medical advice regarding weight gain in pregnancy (including adherence to the Institute of Medicine (IOM) guidelines) (IOM (Institute of Medicine) and NRC (National Research Council, Weight Gain During Pregnancy: Re-examining the guidelines, 2009)), the addition of daily weighing would provide a low cost and simple intervention to reduce excessive weight gain in pregnancy by maintaining weight gain within the target range.

**Methods:**

Women presenting for antenatal care to a secondary level hospital were randomised to routine care or daily weight monitoring. Both groups received nutrition and exercise advice.

**Results:**

Three hundred and ninety-six women were randomised to either the daily weight monitoring group or control group with complete data available for 326 women. The percentage weight gain above target (86.9% (SD 52.3) v 92.7% (SD 50.8) *p* = 0.31) and change in weight per week during the study period (0.59 kg (SD 0.30) v 0.63 kg (SD 0.31) *p* = 0.22) were lesser in those undergoing daily weighing compared to routine management, however these did not reach statistical significance.

**Conclusion:**

Daily weight monitoring as a stand-alone intervention has potential to reduce excessive gestational weight gain. It may have a role as a part of a larger intervention involving dietary and exercise modifications.

**Trial registration:**

The trial was prospectively registered with the Australian New Zealand Clinical Trials Registry. (ACTRN12613001165774, 23/10/ 2013).

## Background

Gestational weight gain is a modifiable risk factor for pregnancy and post pregnancy complications. The short term complications of excessive gestational weight gain (GWG) include fetal macrosomia and increased caesarean delivery rates [1], as well as early childhood obesity [2]. In the longer term, the offspring of mothers who have excessive GWG have an earlier average age of menarche and a greater proportion measure as obese during their pre-schooling, schooling and adult years compared to peers born to mothers who were able to adhere to GWG recommendations [3–5]. For the woman herself, excessive GWG is associated with postpartum weight retention and lifetime obesity [6].

Although previously part of routine practice for every antenatal visit [7], current Royal Australian and New Zealand College of Obstetricians and Gynaecologists guidelines for routine antenatal care and management suggest weighing a pregnant woman only at her booking visit, in order to determine her pre pregnancy body mass index (BMI) and stratify risk [8]. Guidelines for antenatal care in women with obesity suggest monitoring weight gain regularly in pregnancy but do not suggest a frequency [9]. Whilst an increased frequency of weight monitoring in pregnancy has not been proven to improve maternal or fetal outcomes, multiple studies have not been consistent in practice, and have focused on weighing at routine antenatal clinic appointments [10, 11], monthly [2], or at the determination of the woman [12].

Longitudinal studies involving non pregnant populations have demonstrated that more frequent weight monitoring is associated with improved appropriation of behaviours likely to effect weight control positively [13]. This affirmative effect has been evident even in those under acute, short-term stressors associated with a tendency towards weight gain [14]. Daily weight monitoring has been demonstrated to be an effective adjunct management strategy for the prevention of weight gain in young adult women up to the age of thirty-five [15], an age range which encompasses almost 80% of the women giving birth in Australia [16].

The purpose of this study was to assess whether in addition to standardized medical advice regarding weight gain in pregnancy (including adherence to the Institute of Medicine [IOM] guidelines) [17], the addition of daily weighing would provide a low cost and simple intervention to reduce excessive weight gain in pregnancy by maintaining weight gain within the target range.

## Methods

### Trial design

A non-blinded parallel randomized controlled trial with equal allocation to control and intervention groups.

### Eligibility criteria and setting

Women in the second trimester of a singleton pregnancy booking in to deliver in an outer metropolitan hospital in Queensland (Redcliffe Hospital) were considered for enrolment. Exclusion criteria included poor English proficiency, multiple pregnancy, previous bariatric surgery, pre-existing medical disease including diabetes, hypertension or renal disease and smoking. Potential participants were identified at their booking in visit with a midwife or doctor and provided with written information outlining the study process and rationale. Any medical practitioner within the clinic completed enrolment into the study and written informed consent was obtained. Patients were then randomised to the two treatment groups using sequentially numbered opaque envelopes [18]. The actual randomisation category was concealed in the envelope. The lead researcher generated the random allocation sequence and all researchers in the study enrolled participants and assigned them to the treatment group.

### Interventions

The women allocated to the control group received written and verbal information regarding appropriate weight gain in pregnancy [19, 20]. IOM guidelines regarding weight gain in pregnancy are explicitly displayed in the standardized Queensland Health pregnancy hand held health record provided to all women undertaking public hospital led antenatal care in Queensland and these were discussed with the women enrolled (as per standard practice). Women allocated to the treatment group were provided with the same information as controls, but were additionally provided with a set of digital scales and a weight diary with instruction to record their weight each day. However, these scales were provided as an incentive to join the trial only, and were not standardized.

### Outcomes

The primary outcome was percentage weight change above target range, that is, change in weight in kilograms during the pregnancy divided by the top of the target range in kilograms (defined by the IOM guidelines) multiplied by 100. The top of the target weight gain range during pregnancy was 18 kg for those underweight, 16 kg for those in a healthy weight range, 11.5 kg in those overweight and 9 kg for those who are obese. A value above 100% would indicate that on average the change in weight during pregnancy was greater than the expected target range. The secondary outcomes were change in weight in kilograms per week (change in weight per week during the study period = (final weight-booking weight)/total weeks between booking and final weighing) and proportion of women above the target range as defined by the IOM guidelines and described above. Routine perinatal data for neonatal biometric parameters, neonatal wellbeing and the development of maternal pregnancy related medical conditions were also collected and described. Neonatal resuscitation and analgesia were considered binary variables with no differentiation between method intensity.

### Sample size determination

The sample size calculation was based on the percentage weight change above target range for a mean difference of 10% and an estimated standard deviation of 30%. Using a t-test with alpha level of 5, 80% power and equal allocation to study groups, 142 patients were required in each arm. A 15% correction was applied to account for potential non-normal distribution of the outcome variable (allowing analysis by a Mann-Whitney U test), equating to a minimum of 164 patients required in each group. Recruitment of 200 patients per arm of the study was allowed to account for loss to follow up and withdrawal from the study.

### Statistical methods

Statistical analysis was performed with SPSS version 22 (IBM Corp, Armonk, NY) and an intention-to-treat analysis of data was undertaken as adherence to protocol data was not collected. The comparison of interest was between the control and intervention treatment groups. Linear and logistic regression analyses were done on baseline patient characteristics to ensure that those patients excluded from analysis (see CONSORT) did not belong to a population that differed from those included in the study. Continuous variables were examined using a Student t-test or Mann-Whitney U test if not normally distributed. Categorical variables were examined using the Pearson’s Chi-Squared test and Fisher’s Exact test was used when more than 20% of the expected counts were less than 5. For the proportion of women above target range variable the 95% Wald confidence intervals were reported. Descriptive statistics are given for the three outcomes by BMI category.

## Results

**Fig. 1:**
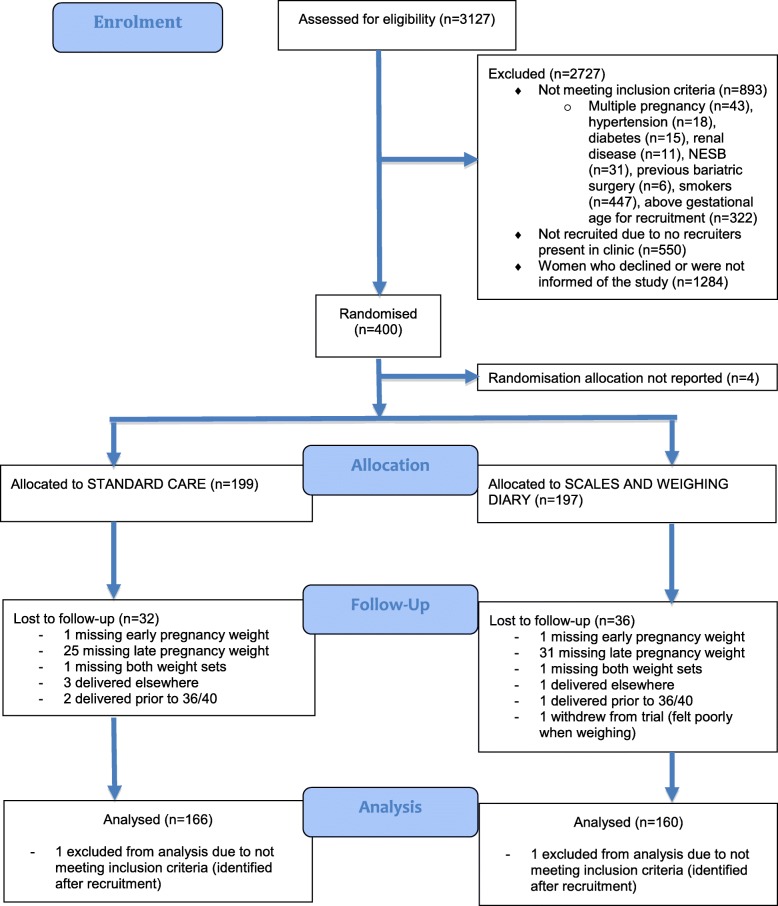
CONSORT Flow Diagram

Recruitment for the study commenced in December 2013 and was completed in October 2015. Of the 400 women enrolled into the study, seventy-four women in total were excluded from the final analysis with the majority (*n* = 60) excluded because of missing or inadequate data (including one withdrawal of consent). Of these, seven were excluded due to a pre-term delivery or delivery at a different facility. Four had not received a group allocation and one intervention patient withdrew from the trial (Fig. 1).

Those women lost to follow-up had similar characteristics to those included in the study, as shown in supplementary Table 1. Two patients were excluded from the final analysis, one from each arm of the study as they were identified after recruitment as being smokers (an exclusion criteria).

Patients included in the study had complete data for weight at booking and a weight at delivery or 36 weeks if not at delivery. Therefore, valid data was available for 326 women, of which, 166 (50.9%) women belonged to the control group and 160 (49.1%) women belonged to the experimental group.

Demographic data is summarised in Table 1 and demonstrates that the two groups had similar baseline characteristics with no statistical differences seen. The mean total number of weeks between the first and last weighing was 18.0 (SD 2.2) for the control and 17.8 (SD 2.3) for the intervention group, and was not statistically significantly different (*p* = 0.32).

**Table 1:** Maternal demographics by treatment group

Demographic variables	Control (***n*** = 166)	Intervention (***n*** = 160)
	n (%)	n (%)
Born in Australia	123 (74.1%)	121 (75.6%)
Aboriginal and Torres Strait Island Status	3 (1.8%)	5 (3.1%)
Age (years, mean (SD))	29.2 (5.5)	28.6 (5.5)
Parity (n = 325)		
Null	53 (32.1%)	53 (33.1%)
1	55 (33.3%)	62 (38.8%)
2+	57 (34.5%)	45 (28.1%)
Previous miscarriages	46 (27.7%)	45 (28.1%)
Previous termination of pregnancy (n = 325)	23 (13.9%)	12 (7.5%)
Gestational age at booking (mean (SD))	20.8 (1.7)	21.0 (1.6)
Weight at booking (mean (SD))	74.7 (18.1)	74.7 (16.7)
Body mass index at booking (mean (SD))	27.2 (6.1)	27.8 (5.9)
Body mass index at booking		
Underweight/healthy (< 25)	66 (39.8%)	66 (41.3%)
Overweight (25–29)	62 (37.3%)	44 (27.5%)
Obese (30+)	38 (22.9%)	50 (31.3%)

The average percentage weight change above the target range for the control group was 92.7% (SD 50.8) compared to 86.9% (SD 52.3) in the intervention group, as shown in Table 2. These high percentages indicate that on average total GWG is within the accepted ranges for both groups. On average women in the intervention group have a lower percentage weight change above the target range compared to the control group, a mean difference of 5.8% (95% CI -5.4 – 17.0). This difference suggests lower total GWG in the intervention group however the difference is not statistically significant (*p* = 0.31).

**Table 2:** Comparison of maternal and neonatal clinical outcomes by treatment group

Characteristics	Control (n = 166)	Intervention (n = 160)	***p***-value
	n (%)	n (%)	
**Maternal clinical characteristics**			
Gestational diabetes	25 (15.1%)	33 (20.6%)	0.19
Hypertension or pre-eclampsia	8 (4.8%)	8 (5.0%)	0.94
Gestation at delivery (days, n = 325, median (IQR))	276 (272–283)	276 (270–283)	0.40
Method of delivery			0.80
Vaginal	105 (63.3%)	99 (61.9%)	
LSCS	61 (36.7%)	61 (38.1%)	
Estimated blood loss for a vaginal birth (mL, *n* = 202, median (IQR))	200 (150–338)	225 (150–400)	0.70
Estimated blood loss for a LSCS (mL, *n* = 122, median (IQR))	400 (300–600)	500 (350–600)	0.33
Percentage weight change above target range^a^ (mean (SD))	92.7 (50.8)	86.9 (52.3)	0.31
Change in weight per week during the study period (kg/wk., mean (SD))	0.63 (0.31)	0.59 (0.30)	0.22
Proportion above the target range	61 (36.7%)	50 (31.3%)	0.30
**Perinatal clinical characteristics**			
Weight (g, n = 325, mean (SD))	3531 (489)	3458 (480)	0.17
Length (cm, *n* = 323, mean (SD))	51.4 (2.6)	51.0 (2.4)	0.15
Head circumference (cm, n = 325, median (IQR))	35 (34–36)	35 (34–36)	0.76
APGAR 1 min (*n* = 325, median (IQR))	9 (8–9)	9 (8–9)	0.95
APGAR 5 min (*n* = 322, median (IQR))	9 (9–9)	9 (9–9)	0.80
Admission to Special Care Nursery (*n* = 324)	43 (26.1%)	48 (30.2%)	0.41
Length of stay in SCN (days, *n* = 90, median (IQR))	3 (2–4)	2 (2–3)	0.47
Fetal distress	31 (18.7%)	36 (22.5%)	0.39

^a^Percentage weight change above target range = change in weight in kilograms during the pregnancy divided by the top of the target range in kilograms (defined by the IOM guidelines) multiplied by 100. A value above 100% would indicate that on average the change in weight during pregnancy was greater than the expected target range

There were no significant differences in BMI or BMI category between the two groups at booking. Table 3 presents descriptive statistics for the three outcomes by BMI category. Those women in the overweight category demonstrated the greatest difference between control and intervention groups with regards to both percentage weight change above target (104.1% (SD 42.2) v 93.6% (SD 50.4), and change in weight per week (0.66 kg/wk. (SD 0.27) v 0.61 kg/wk. (SD 0.32) with lesser weight gain demonstrated in those in the intervention group. Overall, 110 (33.7%) women were below target weight gain, 105 (32.2%) within target range and 111 (34.0%) above target range.

**Table 3:** Descriptive statistics of the three-outcome variables by BMI at booking and treatment group

	% weight change above target range		Change in weight (kg) per week		Proportion above target range	
	Control	Intervention	Control	Intervention	Control	Intervention
	mean (SD)		mean (SD)		n (%)	
Body mass index at booking						
Underweight/healthy (< 25)	76.5 (33.2)	72.9 (28.6)	0.68 (0.28)	0.64 (0.22)	15 (24.6%)	9 (18.0%)
Overweight (25–29)	104.1 (42.2)	93.6 (50.4)	0.66 (0.27)	0.61 (0.32)	27 (44.3%)	18 (36.0%)
Obese (30+)	102.4 (76.4)	99.6 (71.4)	0.50 (0.37)	0.51 (0.36)	19 (31.1%)	23 (46.0%)

The average change in weight per week between randomisation and delivery for women in the control group was 0.63 kg (SD 0.31) and 0.59 kg (SD 0.30) for the intervention group, a mean difference of 0.04 kg (95% CI -0.02 – 0.11) which was not statistically significant (*p* = 0.22, Table 2). There was no association between the proportion of women above the target range and treatment group with 36.7% (95% CI 0.29–0.44) of women above the range in the control group and 31.3% (95% CI 0.24–0.38) in the intervention group (*p* = 0.30).

There were no significant differences in the development of gestational diabetes (15.1% v 20.6%, *p* = 0.19), gestational hypertensive conditions (4.8% v 5.0%, *p* = 0.94), median gestational age at delivery (276 days v 276 days), mode of delivery (*p* = 0.80) or blood loss between the control and intervention groups (vaginal *p* = 0.70, LSCS *p* = 0.33), as shown in Table 2. There were also no significant differences with regards to neonatal weight/size parameters (weight: 3531 g v 3458 g *p* = 0.17, length: 51.4 cm v 51.0 cm *p* = 0.15), or markers of neonatal wellbeing (intervention for suspected fetal distress 18.7% v 22.5% *p* = 0.39, median APGAR scores at 1 min 9 v 9 *p* = 0.95, median APGAR scores at 5 min 9 v 9 p = 0.80, admission to special care 26.5% v 30.2% *p* = 0.41).

## Discussion

This study aimed to determine whether a simple and low-cost intervention, namely daily weight measurement, could be effective at reducing excessive GWG. Unexpectedly, women in both the intervention arm and the treatment arm as a group were adherent to gestational weight gain recommendations, and excessive weight gain was not demonstrated. Our results did demonstrate that daily weight monitoring did not ameliorate GWG to statistical significance in comparison to standard care however there was a trend towards lesser weight gain in the cohort assigned to daily weighing. Although not powered to examine the secondary outcomes in depth there were no demonstrated differences in the development of obstetric medical conditions or labour complications. Similarly, there were no differences in measured offspring outcomes.

Our findings are consistent with previous research in this area. Some studies have demonstrated that weighing at all antenatal visits, monthly and at patient determined frequencies are inadequate as standalone interventions at ameliorating excessive GWG [10–12]. In contrast to these, Jeffries et al. have reported that regular weight monitoring is effective at minimizing excessive GWG in women who are overweight [2]. Similarly, in our trial, the data suggests that those women identified as overweight in the intervention group had lower weight gain compared to the controls, and these results were greater than those seen in normal or obese BMI. Both this trial and that of Jeffries et al. however these are small, single centre trials and larger multi-centred trials may confirm or refute the strength of this association.

Measures that have been shown to mitigate excessive weight gain in pregnancy include dietary and exercise modifications that are often resource intensive and costly [21, 22], and as such may not be appropriate to apply to the general pregnant population. Targeted application of any proven, effective intervention may help limit financial impact and so identification of groups likely to benefit is of paramount importance. This study and that of Jeffries et al. suggest that this target group may be those who are overweight and the benefits of prevention of excessive gestation weight gain for the woman include lesser postpartum weight retention and lifetime obesity [6], and for their offspring, prevention of fetal macrosomia [1], early childhood obesity [2], and adolescent and adult obesity [3–5]. Demonstration of adherence to guidelines (or otherwise) with frequent weight monitoring provides an opportunity for targeted instigation or reinforcement of measures that have been shown to help prevent excessive GWG. Although we did provide women with a simple diary in which to record their home weight measurements we deliberately chose not to keep or analyse any of that data. We have previously observed that paperwork ancillary to the routine pregnancy health record (such as blood sugar monitoring diaries) was not being brought to clinic appointments or birthing limiting our ability to collect the data, and as we were aiming to improve intrinsic motivation and as much as practicable limit medical and midwifery identification of treatment group status we did ask to view diaries at appointments.

Objections have previously been raised against frequent weighing in pregnancy principally on the grounds that it detects neither small for gestational age babies nor pre-eclampsia, and may result in unnecessary maternal anxiety [7]. As such guidelines for antenatal assessment in the United Kingdom recommend weighing after the booking visit only if it is going to impact upon clinical management [23]. Recent research has highlighted the negative impact of excessive gestational weight gain in pregnancy by significantly increasing the risk of both a woman and her offspring becoming overweight or obese [4, 5, 24]. Furthermore, the greater the weight gain in a first pregnancy, the greater is the woman’s pre-pregnancy weight in a subsequent pregnancy affecting the risk factor profile for that pregnancy [25]. Therefore, clinical management should be altered in any woman demonstrating increased gestational weight gain in an effort to inhibit further excessive gain. Identification and moderation of excessive weight gain is then an important obstetric consideration with a public health impact extending beyond immediate pregnancy complications.

We chose to encourage very frequent monitoring of weight despite the previously stated concerns regarding a negative mental health impact as prospective trials in both pregnant and in non-pregnant populations have failed to demonstrate any negative consequences associated with daily weight monitoring [26–29]. Conversely, participants demonstrated less depressive symptoms and anxiety [26], and improved control with regard to behaviours that may impact upon weight maintenance [27]. Additionally, when aware of the rationale for weight gain recommendations, women have expressed a preference for frequent weight monitoring [28]. We did not assess the acceptability of our intervention to the subject group, nor their emotional responses to weighing themselves daily and as such can’t be certain that it did cause some negative emotional side effects that would potentially limit its utilisation.

Although our cohort was representative of women routinely presenting for antenatal care at our hospital and the intervention is available to most people without great expense, the generalisability of our findings may be limited by our cohort as the lesser rates of weight gain seen in both groups may indicate a higher degree of health literacy than usually seen in the population. Smoking was one of the initial exclusion criteria, as such, some women in a lower socio-economic group in the community, as well as lower education levels, may have been excluded from the study. A major limitation of the study was that adherence to study protocol was not assessed and this may have contributed to the small differences in the outcome measures observed between the study groups. Furthermore, although the average age at delivery was unchanged by group allocation, those delivering prior to 36 weeks were excluded from analysis. Overall, there was a 17.0% loss to follow up which is significant however a sensitivity analysis showed no statistical differences between baseline characteristics of those lost to follow up from those who completed the trial. Our sample size calculation was an underestimate as the standard deviation observed was greater than expected and the effect size of the effect of the treatment smaller than anticipated for our primary outcome. This means that this study was not powered to detect the size of the effect we were anticipating but it does provide estimates for future studies and suggests that the daily weighing has potential to contribute to reducing GWG. Our secondary outcomes could be examined in further studies as they too suggest GWG can be reduced with daily weighing but the size of the difference between groups is small. Finally, recruitment in the mid second trimester may have been too late for our intervention to have an impact especially given that excessive first trimester weight gain is related to childhood obesity and the development of maternal gestational diabetes [30, 31]. In order to provide greater information regarding potential harms, as well as successful adjuncts to this intervention, information regarding dietary intake including meal frequency and composition, as well as psychological and emotional wellbeing during the trial period should also be assessed.

## Conclusion

Our study indicates daily weight monitoring alone has the potential to reduce GWG. Although we demonstrated some evidence of a reduction in excessive weight gain in pregnant women who have been given advice to weight themselves daily, this reduction was not as great as we had anticipated. Further studies are needed to determine if there is a benefit from this intervention in targeted BMI categories or in addition to appropriate dietary and exercise interventions. Further investigations into preterm births and the impact of gestational weight gain would also be beneficial.

## Supplementary information

**Additional file 1 Supplementary Table 1.** Maternal demographics by treatment group for those women lost to follow-up.

## Data Availability

The datasets used and/or analysed during the current study are available from the corresponding author on reasonable request.

## Abbreviations

GWG: Gestational weight gain
BMI: Body mass index
IOM: Institute of Medicine
