# Supplementary material for: A randomized controlled trial of daily weighing in pregnancy to control gestational weight gain
Source: BMC Pregnancy Childbirth. 2020 Apr 16;20:223. doi: 10.1186/s12884-020-02884-1 (PMC7164155; doi:10.1186/s12884-020-02884-1)
Supplement: Supplementary file 1 — Additional file 1 Supplementary Table 1. Maternal demographics by treatment group for those women lost to follow-up. [file 12884_2020_2884_MOESM1_ESM.docx]

**Supplementary Table** 1: Maternal demographics by treatment group for those women lost to follow-up.

| **Demographic variables** | **Lost to follow-up** | |
| --- | --- | --- |
|  | **Control** | **Intervention** |
|  | **(n=32)** | **(n=36)** |
|  | **n (%)** | **n (%)** |
| Born in Australia | 27 (87.1 %) | 26 (74.3 %) |
| Aboriginal and Torres Strait Island Status | 4 (12.9 %) | 2 (5.7 %) |
| Age (years, mean (SD)) | 27.7 (5.8) | 26.4 (5.9) |
| Parity |  |  |
| Null | 13 (41.9 %) | 19 (54.3 %) |
| 1 | 8 (25.8 %) | 11 (31.4 %) |
| 2+ | 10 (32.3 %) | 5 (14.3 %) |
| Previous miscarriages | 6 (19.4 %) | 7 (20.0 %) |
| Previous termination of pregnancy | 7 (22.6 %) | 2 (5.7 %) |
| Gestational age at booking (mean (SD)) | 20.7 (1.8) | 20.7 (1.2) |
| Weight at booking (mean (SD)) | 67.2 (17.4) | 69.7 (16.4) |
| Body mass index at booking (mean (SD)) | 23.7 (7.2) | 25.0 (5.0) |
